# Description of larva and pupa of *Laenahaigouica* (Tenebrionidae, Laenini) from China based on morphology and four DNA makers

**DOI:** 10.3897/zookeys.852.32095

**Published:** 2019-06-05

**Authors:** Zhonghua Wei, Guodong Ren

**Affiliations:** 1 The Key Laboratory of Zoological Systematics and Application, Hebei University Baoding China; 2 College of Life Sciences, Hebei University Baoding China; 3 Hebei University, 071002, Baoding, Hebei Province, China Hebei University Baoding China

**Keywords:** DNA markers, *
Laena
*, larva, pupa, darkling beetle, systematics

## Abstract

The larvae, pupae, and adults of *Laenahaigouica* Schawaller, 2001 were collected during recent fieldwork in the Sichuan Province (China). Since the pupal morphology of *Laena* has never been investigated this created an opportunity to provide the first description. Moreover, prior to this study larval characters of only one species, *Laenastarcki* Reitter, 1887, were known. Therefore, description of the larva of *L.haigouica* enabled the first verification of the intrageneric stability of larval characters revealed for other *Laena* species. Association of the studied immature stages with the adults was confirmed by analysing COI sequences. Additionally, three other loci (16S, Cytb, 28S) were sequenced for *L.haigouica* during this study.

## Introduction

The genus *Laena* contains approximately 261 species in Palaearctic region (Schawaller 2008). The larva of *Laenastarcki* Reitter, 1887 was first described by Byzova (1958). Watt (1974) stated that he examined the larvae of *Laenaviennensis* Sturm, 1807 from Slovenia, but he did not provide a detailed description. In that paper, Watt also corrected the mistaken description of Byzova, which was that the antennae of *Laena* larvae have two segments not three segments. However, the authors of this study demonstrated that antennae of *Laena* larvae with three segments (Fig. 2D). *Laena* larvae have a body shape of the tenebrionid type, like wireworms. Knowledge of their morphology, development and habits is very scarce. Doyen (1988) estimated that there are approximately 240 genera and 300 species of tenebrionid larvae described. Recently, many pupae of tenebrionid beetles were described (Steiner 1995; Bouchard and Steiner 2004; Iwan and Schimrosczyk 2008; Purchart and Nabozhenko 2012; Wagner and Gosik 2016; Kamiński et al. 2018). Kamiński et al. (2018) summarized the known data on pupae of the ‘Opatrinoid’ clade and provided a checklist. However, few pupa of Lagriinae have been described, including *Lagriavillosa* Fabricius, 1781 described by Spilman (1978) and pupa of *Centorusprocerusmoldaviensis* Reitter, 1920 described by Cherney (2005). Of the *Laena* species, only one species has larval stages described, and no pupal stages described. The morphology of larvae of the genus *Laena* has been barely dealt with, and only two species have been utilized for phylogenetic studies (Aalbu et al. 2017).

Recently, the larvae, pupae, and adults of *Laenahaigouica* Schawaller, 2001 were collected during fieldwork in damp deadwood in Sichuan Province of China. Therefore we speculated that the larvae of *L.haigouica* were feeding on deadwood or fungi. The larva and pupa are described, photographed, and figured for the first time in this paper.

## Materials and methods

Larvae, pupae and adults of *Laenahaigouica* were collected on 27 July 2016 from Zhongcha rangeland, alt. 2870 m, Jiuzhaigou County (Sichuan Province, China) by Xiumin Li, Xinglong Bai, Xianlei Shao and Runyang Zhang. All examined specimens were preserved in 70% alcohol and deposited in the Museum of Hebei University, Baoding, China.

Larvae were observed and described using Nikon SMZ800. Photographs of larvae and pupae were taken with a desktop SEM Hitachi TM3000 and Leica M205A stereomicroscope equipped with a drawing tube, and a Leica DFC450 camera.

Total genomic DNA was extracted from larval, pupal, and adult tissue using EZNA Insect DNA Kit (Omega Bio-tek, USA), following manufacturer’s protocols. One fragment of the mitochondrial protein-coding gene (COI) was amplified respectively from larva, pupa, and an adult; one fragment of the mitochondrial protein-coding gene (Cytb) was amplified from an adult; one fragment of the mitochondrial ribosomal RNA gene (16S) was amplified from an adult; and one fragment of nuclear rRNA gene (28S) was amplified from an adult. The detailed methods of the molecular studies are the same as those used in Li et al. (2018). Sequences were aligned using the ClustalW algorithm (Thompson et al. 1994) as implemented in BioEdit 7.0.9.0. (Hall 1999).

## Taxonomy

### Laena haigouica

Taxon classificationAnimaliaColeopteraTenebrionidae

Schawaller, 2001

Laena haigouica Schawaller, 2001: 19–20, figs 41–44. Type locality: China, Sichuan, Jiuzhaigou.

#### Specimens examined.

Larvae (11 ex), pupae (2 ex), adults (4♂7♀), Sichuan, Jiuzhaigou, Zhongcha rangeland, 2870 m, 27.VII.2016, Xiumin Li, Xinglong Bai, Xianlei Shao & Runyang Zhang leg., HBUM.

#### Larva.

***Diagnosis.*** The larva of *L.haigouica* Schawaller, 2001 can be separated from *L.starcki* Reitter, 1887 by following characters: clypeus transverse, 3.3 times wider than long, surface with four long erect setae; abdominal spiracles on lateral margins of tergites III–VIII in middle or just before middle.

**Figure 1.**
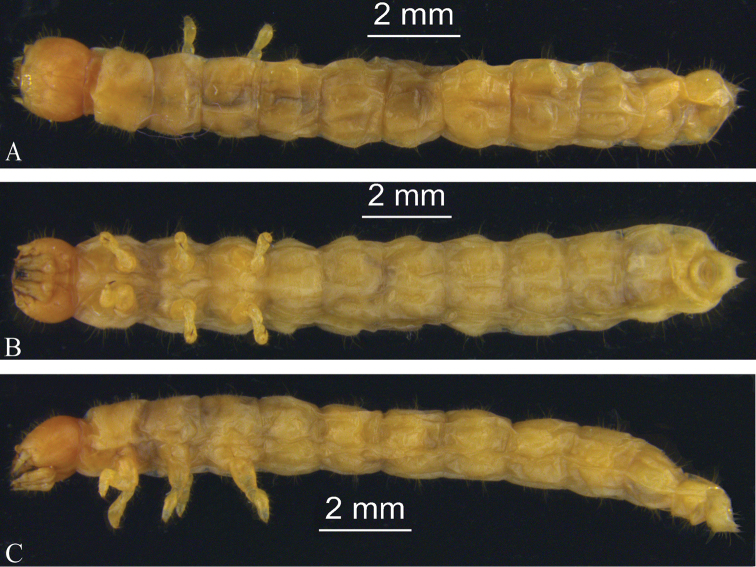
Larva of *Laenahaigouica* Schawaller, 2001 **A–C** habitus, in dorsal, ventral, and lateral views.

***Description.*** The description is based on what is probably a later instar larva. Body length 15–17 mm. Body (Fig. 1A–C) elongate, parallel-sided, subcylindrical; setose; integument soft; white to light brown in colour; thoracic and abdominal segments subcylindrical. Abdomen without defensive glands.

**Figure 2.**
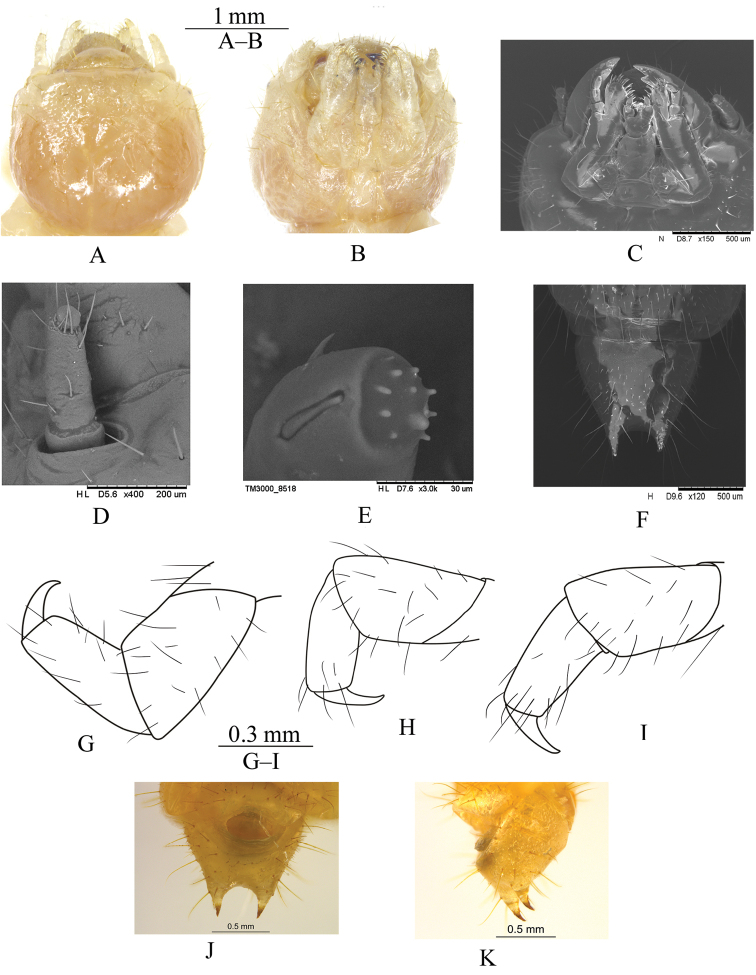
Larval morphology of *Laenahaigouica* Schawaller, 2001 **A–B** head, in dorsal and ventral views **C** mouth and appendages, in ventral view **D** antenna, in lateral view **E** apex of maxillary palpus **F** segments IX, in dorsal view **G–I** pro-, meso- and metalegs, in ventral view **J–K** urogomphi in ventral and lateral views.

***Head.*** (Fig. 2A–B). Light brown. Weakly declined; width slightly narrower than prothorax; distinctly constricted before occipital foramen; sides rounded; punctation minute, dense, separated by 1–3 puncture diameters. Epicranial suture stem length approximately one-half head capsule length; frontal arms Y-shaped. Frons smooth. Epicranial plates light brown, smooth, with sparse short and few long erect setae; lateral portion with both short and long erect, setation denser than dorsal part; ventral portion of each plate with three long erect setae and sparse short setae. One small stemmata present on each epicranial plate, before Y-shaped frontal arms. Labrum transverse, widest in middle, surface convex, with six to seven long erect setae; anterior margin weakly protruding; lateral margins curved, contracted at apical part. Clypeus transverse, surface convex, with four long erect setae. Mandible well developed, with two small teeth on anterior inner sides. Maxillary palpi cylindrical, apex (Fig. 2E) with nine sensilla knobble. Ligula (Fig. 2C) apex with two long erect setae. Mentum longer than wide, widest in middle. Anterior margin of gula distinctly narrower than posterior margin. Antenna (Fig. 2D) short than head, tri-segmented, clavate; second segment longer than first, third segment small and rounded, prominent, surrounded by seven long erect setae.

***Thorax.*** Thoracic tergites light yellow. Prothoracic tergum subquadrate, longer than wide, 1.4–1.5 times as long as meso- or metatergites; surface with short and long erect setae. Meso- and metatergites wider than long; surface with short and long erect setae. Spiracles oval; prothoracic and metathoracic without spiracle; mesothoracic spiracles on anterolateral part, largest, approximately twice size of abdominal spiracles. Metathoracic tergum distinctly wider than pro- and mesothoracic tergum. Coxal cavity distinctly separate.

***Abdomen.*** Abdominal tergites light brown, slightly darker than sternites; surface smooth, with short and long erect setae. Abdominal tergites I–VIII wider than long, widest in middle; lateral margins of abdominal tergites curved. Abdominal tergite IX distinctly narrower than tergite VIII; anterior margin distinctly wider than posterior margin; tergite IX posteriorly round, armed with pair of acute urogomphi (Fig. 2F, J, K). Abdominal spiracles on lateral margins of tergites III–VIII in middle or just before middle.

***Legs.*** Surface with long erect setae; proleg (Fig. 2G) distinctly longer, slightly thicker than meso- and metalegs (Fig. 2H–I); tarsungulus sickle-shaped, prothoracic tarsungulus more sclerotized than meso- and metathoracic tarsungulus; prothoracic trochanter shortest and thickest; posterior trochanter short and thick; prothoracic tibia curved, slightly longer and slender than meso- and metathoracic tibia.

#### Pupa.

***Diagnosis.*** Compared to the known pupa of *Lagriavillosa* described by Spilman (1978) and pupa of *Centorusprocerusmoldaviensis* described by Cherney (2005) in subfamily Lagriinae, the pupa of *Laena* can be easily separated by light color, sparse setae, urogomphi 0.7 (n=2) times length of tergite IX, lateral margins of abdominal segments I–VII each with tubercle bearing long erect setae, abdominal tergites I–VIII with tubercles.

***Description.***

**Figure 3.**
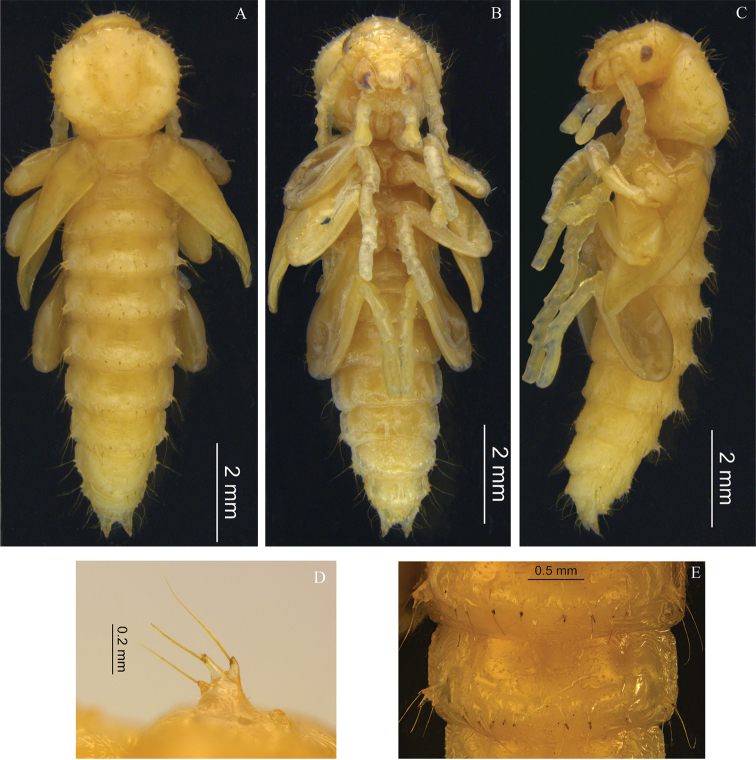
Pupal morphology of *Laenahaigouica* Schawaller, 2001 **A–C** habitus, in dorsal, ventral and lateral views **D** tubercles of lateral margins of abdominal segments **E** setae of abdominal tergites.

Body length 10.5–11.2 mm, body width 2.5–2.9 mm; body white to light brown (Fig. 3A–C), with darker apices of spine on urogomphi, with black eyes, and light brown mandible apices; body with sparse long erect setae, setae yellow; abdominal tergites with developed lateral processes bearing two to three long erect setae; abdominal tergum IX with paired upturned urogomphi.

***Head.*** Dorsal surface smooth, concealed (invisible in dorsal view). Anterior margin of labrum rounded, with sparse short setae. Clypeus with two long erect setae on each anterolateral side. Frons and vertex with sparse granules bearing long erect setae. Eye ovate, black. Mandible apices brown. Antenna long and thick; antennomeres IV–XI with small brown spots on apex in dorsal view, without setae.

***Thorax.*** Pronotum transverse. Anterior margin straight, with eight granules bearing long erect setae; anterior angles subrectangular, posterior angles rounded; lateral margins curved, each side with seven granules bearing long erect setae. Disc surface convex, with sparse granules bearing long erect setae. Mesonotum and metanotum distinctly narrower than pronotum, each with four setae posteriorly. Mesoventrite, metaventrite and elytra glabrous.

***Abdomen.*** Abdominal spiracles ovate. Tergites I–VIII each with four pairs of setae (Fig. 3E). Lateral margins of abdominal segments I–VII each with tubercle (Fig. 3D) bearing two to three long erect setae, VIII and IX each with three small tubercles each bearing long erect seta. Tergites I–VII transversely convex in middle. Tergite IX posteriorly rounded, with pair of apically sclerotized urogomphi bearing one to two setae at base, ventrite with setae denser than tergite. Ventrites V–VI with four long erect setae. Ventrites VII–IX with setae denser than ventrites I–VI.

***Legs.*** Prolegs distinctly longer than meso- and metalegs. Femora covered with sparse erect setae. Tibiae and tarsi glabrous, without setae.

***Comment.*** These two specimens probably represent early stage pupa, which have the body colour light and antenna and tarsi near translucent.

#### DNA markers.

The mtDNA COI sequences respectively from the larva, pupa and adult were identical after sequence alignment. The gene fragments of 16S, 28S, COI, and Cytb are deposited in GenBank with the accession numbers MK227697, MK227698, MK227699 (pupa), MK227700 (larva), MK227701, and MK227702.

## Discussion

The morphological characteristics of larvae of subfamily Lagriinae were summarized by Hayashi (1964) and Matthews et al. (2010). Larvae of *Laena* species resemble those of *Centorus* species in subfamily Lagriinae. These larvae can be distinguished from other larvae of the subfamily Lagriinae by having three-segmented antennae (Byzova 1958; Cherney 2005). The former can be easily differentiated from the latter by following characters: (1) *Laena* species with Y-shaped frontal arms (*Centorus* species with U-shaped frontal arms); (2) *Laena* species with antennomere II distinctly longer than antennomere I, antennomere III very small and rounded (*Centorus* species with antennomere II nearly equal to antennomere I, antennomere III short and columned); (3) *Laena* species with stemmata before Y-shaped frontal arms (*Centorus* species with stemmata behind U-shaped arms); (4) abdominal tergites with tubercles each bearing a long erect seta.

The description provided above for *L.haigouica* is the first contribution to the knowledge on the pupal stages of Laenini. Pupa possess lateral processes which were considered to be plesiomorphic among the whole Tenebrionidae (Steiner 1995). The pupae of *Laena* species can be easily separated from known pupae of *Lagria* species (Spilman 1978) and *Centorus* species (Cherney 2005) in Lagriinae by having a body with sparse long setae and abdominal tergites with tubercles each bearing a long erect seta.

## Supplementary Material

XML Treatment for Laena haigouica
